# Rural first-mile pickup and last-mile delivery: A bus-assisted heterogeneous-drone model

**DOI:** 10.1371/journal.pone.0344897

**Published:** 2026-04-08

**Authors:** Song Jin, Lu Wang, Yunpeng Gong, Jingyu Hu

**Affiliations:** Xi’an University of Architecture and Technology HuaQing College, Xi’an, Shaanxi, China; National Taiwan University of Science and Technology, TAIWAN

## Abstract

With the rapid expansion of rural e-commerce, widely dispersed demand and limited road infrastructure have made conventional truck-based first-mile pickup and last-mile delivery increasingly unsustainable, creating an urgent need for alternative logistics models. We introduce a bus-assisted heterogeneous-drone scheme that treats fixed-route rural buses as mobile hubs while dispatching drones with complementary ranges and payloads for door-to-door service. A mixed-integer programming model captures bus schedules, drone heterogeneity, time-window constraints, and battery limits. To solve this model efficiently, we develop a two-stage framework—bus-stop clustering followed by an Improved Black-Kite Algorithm (IBKA). IBKA incorporates four enhancements: opposition-based learning, adaptive attack probability, random boundary shrinkage, and a Differential Evolution hybrid operator. Numerical experiments on adapted Solomon instances show the proposed method outperforms Gurobi, a standard Genetic Algorithm (GA), an Eel and Grouper Optimizer (EGO), and the original Black-Kite Algorithm (BKA) in terms of cost, stability, and convergence. On average, IBKA reduces total delivery cost by 5% relative to GA, 9% relative to EGO, and 13% relative to BKA, and enhances stability by 23%, 55%, and 23%, respectively. Sensitivity tests highlight the pivotal influence of drone payload and bus headway. A real-world study on the Xunyang–Tongqianguan line in Shaanxi Province further demonstrates substantial cost savings and operational advantages over both truck-only and homogeneous-drone delivery modes, underscoring the practical value of bus–drone collaboration for rural logistics.

## Introduction

The rapid growth of e-commerce has elevated rural logistics into an essential component of the overall logistics industry [1,2]. According to recent reports by the Ministry of Commerce of China and the China Internet Network Information Center, by the end of 2024, China had approximately 313 million rural internet users and 18.532 million rural online businesses, generating online retail sales totaling 2.56 trillion yuan [3,4]. This substantial scale highlights the urgent need to develop efficient logistics models for rural first-mile pickup and last-mile delivery.

Nevertheless, several operational challenges remain in current rural logistics systems. Due to dispersed populations and scattered logistics demands in rural areas, traditional express delivery methods often struggle to maintain efficiency and guarantee high service quality [5,6]. To address these issues, the Chinese government has advocated an integrated approach known as “passenger-cargo-post integration,” aiming to enhance the efficiency and coverage of rural logistics by combining passenger, freight, and postal resources [7]. However, in practice, inadequate transport infrastructure—including unpaved roads in remote villages—limits rural buses primarily to main township routes, creating significant coverage gaps [8].

In this context, integrating drone technology with rural buses to create a coordinated rural first-mile pickup and last-mile delivery model offers a promising strategy to enhance service quality and logistics coverage in rural areas. Drones are flexible, fast, and highly adaptable, enabling precise delivery to remote locations despite challenging terrain and limited accessibility [9]. Conversely, rural buses offer consistent schedules, predefined routes, and access to primary roads, serving effectively as mobile platforms for drone operations—including intermediate landings, battery replacements, and cargo transfers. This integration significantly expands drone operational ranges and alleviates their inherent limitations [10]. The complementary nature of bus and drone operations facilitates resource integration and cost-sharing, substantially enhancing logistics coverage efficiency and establishing a practical foundation for economically viable rural logistics systems.

Existing research predominantly emphasizes the “truck + drone” delivery model, whereas studies exploring coordination between drones and fixed-route, scheduled bus systems remain scarce. A critical challenge involves synchronizing drone operations (take-offs, landings, battery swaps, and cargo handling) with fixed bus schedules without altering established operational patterns [11]. There have been a few studies on bus or rail transit combined with drones, mainly based on urban scenarios with dense demand, centralized stations, and a focus on one-way delivery [12]. In contrast to urban scenarios, rural logistics exhibits widely dispersed demand points, coupled with dual-directional flows consisting of consumer-end deliveries and shipments originating from agricultural products and e-commerce packages [13]. Additionally, rural bus routes are typically sparse and operate at lower frequencies, making it challenging to offer dense and adequate transfer and landing points similar to urban bus systems. Consequently, urban collaborative mechanisms cannot be directly applied to rural contexts, necessitating the development of novel operational and decision-making frameworks tailored explicitly to dispersed rural demands and pickup-delivery scenarios.

To bridge this research gap, this paper proposes a “bus-assisted heterogeneous drone” model tailored explicitly for rural first-mile pickup and last-mile delivery. The study aims to establish a collaborative operational framework capable of managing dispersed demands and bidirectional flows simultaneously, construct an optimization model coordinating bus loading, drone scheduling, and synchronized routing, assess improvements in coverage efficiency, cost, and service quality, and provide actionable decision support for governments and logistics enterprises regarding fleet composition, route planning, and operational management.

The main marginal contributions of this study are summarized as follows.

(1) Compared with existing bus–drone studies that are largely developed for high-frequency urban transit networks and typically focus on one-way last-mile delivery with homogeneous drone fleets, this paper proposes a rural collaborative pickup-and-delivery framework that integrates fixed-route buses with heterogeneous drones. The model captures bidirectional flows and explicitly incorporates two-way fixed bus timetables and stop-based handover/re-boarding mechanisms, thereby representing an operationally implementable collaboration mode under sparse rural transit supply.
(2) To efficiently solve the resulting optimization problem, we develop a two-stage solution framework that combines bus-stop clustering with an Improved Black Kite Algorithm (IBKA). IBKA integrates opposition-based learning, adaptive attack probability, boundary repair, and a Differential Evolution hybrid operator, improving solution quality, robustness, and convergence efficiency.

The rest of the paper is structured as follows: The Literature Review section reviews related work. The Model Formulation section defines the problem and presents the mathematical model. The Solution Method section explains the proposed solution approach. The Numerical Results section report the numerical results. The Case Study section presents the case study. The Managerial Insights section discusses practical implications for decision-makers. The Conclusion section summarises the main findings and outlines directions for future research.

## Literature review

The research problem of this study originates from the concept of Freight on Transit (FoT), which involves simultaneously transporting passengers and cargo using public transit vehicles operating on fixed routes and schedules, thus sharing transport capacity and reducing reliance on dedicated freight trucks [14]. The central challenge of this model lies in coordinating the temporal and spatial linkage between existing public transit trunk routes and last-mile delivery operations. Existing studies typically use buses or rail transit for trunk transportation, supplemented by tricycles or small local vehicles for last-mile delivery, aiming to replace traditional truck-based systems with more energy-efficient and flexible solutions [15–18].

Since Murray and Chu^‌‌^ introduced the “Truck-Drone Delivery Problem,” also known as the Flying Sidekick Traveling Salesman Problem (FSTSP), logistics models integrating vehicles and drones have rapidly gained research attention in operations research and logistics [19]. Initial studies predominantly focused on a “truck-led, drone-assisted” model, scheduling drones to perform deliveries along predefined truck routes [20,21].

As shown in Table 1, with the emergence of the FoT concept, researchers began integrating drones into public transportation systems, exploring collaborative first-mile pickup and last-mile delivery strategies involving buses or rail transit combined with drones. Early studies primarily focused on designing generalized coordination frameworks, in which fixed routes and timetables of buses or rail transit served as trunk transportation, while drones were deployed as complementary tools for last-mile accessibility. In these models, transit stations were utilized as hubs for drone takeoff, landing, battery replacement, and cargo handover. Typical optimization objectives included minimizing total travel time, energy consumption, or distance, subject to basic operational constraints such as drone endurance, payload capacity, and service time windows [22,23].

**Table 1:** Existing literature on bus-/rail-assisted drone delivery.

No	Reference	Model type	Optimization objective	Solution Method	Scenario
1	Lasla et al., 2019	Bus+drone	Minimize energy consumption	MILP exact optimization	City: Simulation cases
2	Huang et al., 2020a	Train+drone	Minimize delivery time	Exact algorithm + Suboptimal Algorithm	City: Simulation cases
3	Huang et al., 2020b	Bus+drone	Minimize round-trip time	Exact DP-based algorithm	City: Simulation cases
4	Huang et al., 2021	Bus+drone	Maximize on-time delivery probability	Label setting algorithm	City: Simulation cases
5	Choudhury et al., 2021	Bus+drone	Maximum completion time	Task allocation + Multi-agent pathfinding	City: San Francisco
6	Deng et al., 2024	Bus+drone	Minimize delivery time	Tabu Search metaheuristic	City: Simulation cases
7	Osorio and Ouyang, 2025	Bus+drone	Minimize total cost	MILP with Lagrangian relaxation and column generation	City: Chicago Metra system

As the research deepened, scholars gradually recognized that different application scenarios entail differentiated challenges and constraints. Consequently, recent studies have proposed more targeted extensions to address the specific characteristics of urban areas. For example, Huang et al. (2020b) incorporated fluctuations in bus capacity during peak hours together with drone endurance limitations, capturing the dynamic coupling between transport capacity and flight range [24]. Huang et al. (2021) further developed a stochastic time-dependent network to systematically analyze the impact of peak-hour congestion on bus arrival times, thereby improving the reliability of route planning [25]. Choudhury et al. (2021), addressing the large scale and high density of urban bus networks, proposed a two-layer framework that decomposes task allocation and path planning, enabling efficient solutions under scenarios involving hundreds of drones and thousands of orders, thus adapting to the scalability requirements of complex urban transit systems [26]. Deng et al. (2024) examined the optimal strategy for drones to either fly directly or ride buses in urban environments, highlighting the trade-offs between energy consumption and delivery time under congested traffic conditions [12].

However, research on the “bus/rail + drone” collaborative model in rural settings remains very limited and has not been systematically explored. Compared with urban environments, rural areas exhibit significant differences in both demand characteristics and transport supply, making it difficult to directly apply existing frameworks that are based on urban assumptions. Specifically:

First, the structure of freight demand differs. In urban areas, where demand volume is large and orders are dense, goods of different weights or sizes can be separated into distinct delivery streams—for example, heavy cargo versus small parcels—such that a homogeneous drone fleet is sufficient to meet service needs. In contrast, rural demand points are scattered and volumes are relatively small, making it more suitable to employ a heterogeneous fleet whose varying payload and endurance capabilities complement each other, thereby improving delivery efficiency.

Second, the direction of logistics flows diverges. Urban distribution primarily targets last-mile delivery of consumer goods, whereas rural areas are not only recipients of consumer products but also important producers and exporters of agricultural products. Consequently, upstream transport demand cannot be neglected, and rural systems must simultaneously optimize bi-directional flows of pickup and delivery tasks, rather than focusing solely on outbound distribution.

Finally, the supply of public transit services differs substantially. In urban settings, public transit networks are dense and service frequencies are high. Thus, in studies of “bus + drone” collaboration, models are often simplified by considering only outbound delivery tasks, with inbound bus trips serving merely as auxiliary resources for vehicle or drone returns. In rural areas, however, routes are sparse, headways are long, and station spacing is large, meaning that both inbound and outbound trips may represent scarce and critical capacity resources. Models must therefore explicitly incorporate bidirectional timetables and feasible connection windows in order to accurately reflect system operability.

In summary, applying the “bus/rail + drone” collaborative model to rural contexts requires targeted modifications and extensions in both model structure and methodological design. In response to this research gap, this study explicitly incorporates heterogeneous fleet configuration, bi-directional pickup and delivery tasks, and inbound–outbound service schedules into the modeling framework, so as to more accurately capture the operational characteristics of rural transport systems.

In terms of solution methods, as shown in Table 1, existing studies on bus- or train-assisted drone delivery mainly adopt three categories of approaches. First, exact optimization methods (e.g., MILP, dynamic programming) can guarantee global optimality, but their computational complexity grows rapidly with the number of demand points, making them impractical for larger problem sizes or more complex constraints. Second, problem-specific heuristics (e.g., round-trip construction, clustering combined with tabu search) improve computational efficiency to some extent, but they typically rely on strong problem-specific assumptions and lack sufficient global search capability. Third, decomposition frameworks (e.g., task allocation with multi-agent pathfinding, Lagrangian relaxation combined with column generation) reduce problem complexity through staged optimization, yet their algorithmic structures are often intricate and may struggle to balance computational efficiency and solution quality when dealing with heterogeneous drones, timetables, and energy constraints simultaneously. Against this backdrop, this study adopts a two-stage framework: in the first stage, demand points are clustered around bus stops to reduce problem size; in the second stage, the Improved Black Kite Algorithm (IBKA) is employed to solve the subproblems. By integrating opposition-based learning, adaptive attack probability, boundary shrinkage, and differential evolution operators, IBKA provides a flexible and adaptive solution approach for bus–drone coordination with heterogeneous fleets.

### Model formulation

#### Problem description.

**Fig 1:**
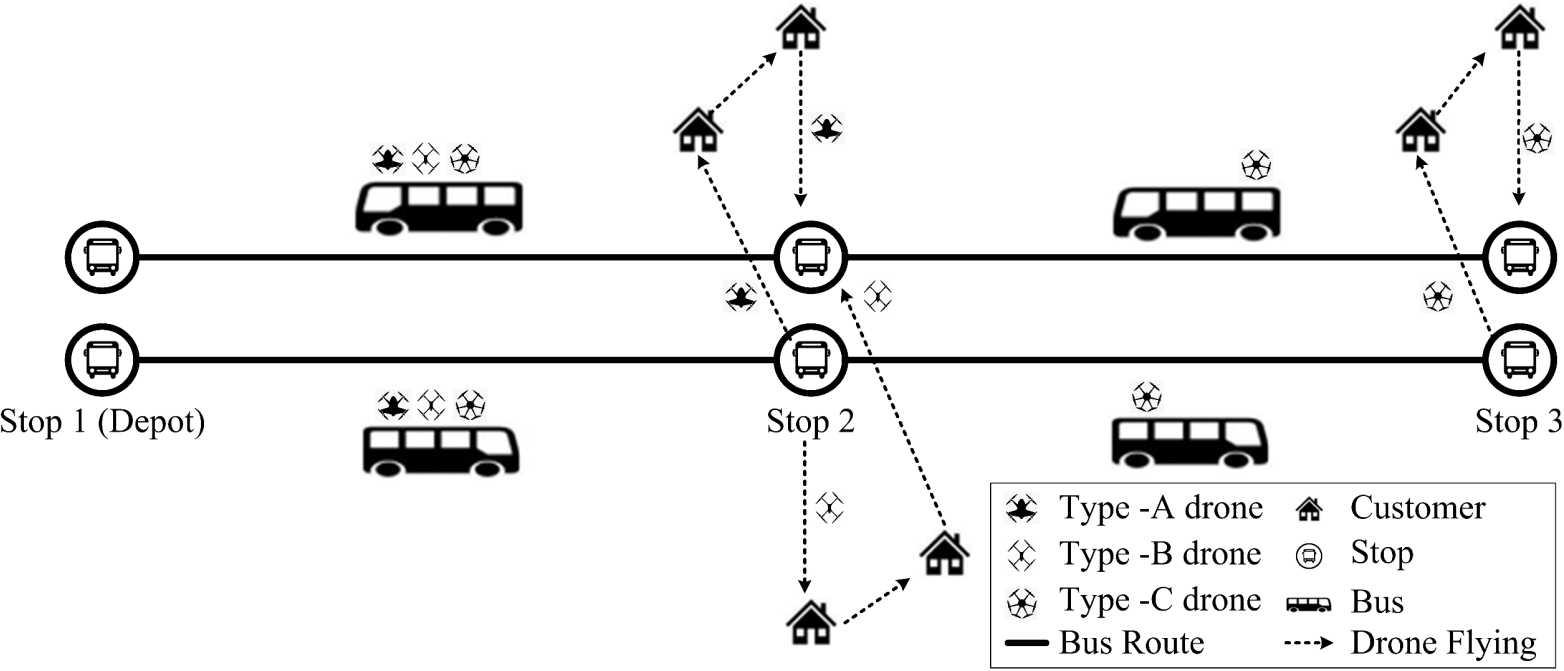
Bus-Assisted Heterogeneous-Drone system representation.

Building on this framework, we now formalize the problem setting and present the mathematical model. In this study, we consider a rural first-mile pickup and last-mile delivery system integrating rural bus operations and drone technology (see Fig 1). Initially, parcels are sorted at a township-level distribution center and subsequently loaded onto rural buses operating on fixed schedules. These buses stop at a series of predetermined stations along their routes. The distribution center also functions as the dispatch and return hub for a heterogeneous drone fleet. Drones can only take off and land at designated bus stops, but they can freely select their flight paths, overcoming road network limitations that traditionally restrict delivery coverage. Each bus allocates a portion of its cargo space specifically for parcel transportation between adjacent stops. The drone fleet comprises multiple drone types, each with varying payload capacities and operational ranges. Drones are capable of performing multiple consecutive delivery and pickup tasks within the constraints of their battery capacity. The operational process is as follows:

- The bus departs from the initial station with drones onboard.
- Drones take off from a specified station to sequentially serve multiple customers according to optimized routes (for delivery, pickup, or both).
- Upon completing each task cycle, drones return to the nearest bus stop for battery replacement, cargo handling (loading/unloading), and task updates.
- If there are remaining unmet demands near the current stop, drones launch again; otherwise, they remain onboard until the next stop.

Through alternating between flying and hitchhiking modes, drones effectively extend their operational range, while buses significantly expand spatial coverage. After completing daily assignments, all drones return to the distribution center via buses for maintenance, thus creating a closed-loop rural logistics system.

### Notations

The detailed notations and descriptions are given in Table 2.

**Table 2:** Notations.

Symbol	Description
Parameters	
S	Set of bus stops
C	Set of customer locations
N	Set of all nodes (including depot, bus stops and customer locations)
K	Set of drone types
$M_{k}$	Set of drones of type k
T={1,...,t}	Index of drone trips (each trip: stop → multiple customers → stop)
$R_{s}=\{1,...,r\}$	Set of bus departures available at stop s
$H^{max}$	Maximum number of drones that can be carried simultaneously by a bus
o, e	The origin and end nodes of each route
$d_{ij}^{d}$	Aerial distance between nodes i and j
$d_{ss\text{'}}^{b}$	Distance between bus stops s and s'
$d_{i}^{pkg}$	Delivery parcel weight to customer i
$q_{i}^{ret}$	Pickup parcel weight from customer i
$Q_{k}$	Maximum payload of drone type k
$R_{k}$	Maximum flight range of drone type k
$v_{k}^{d}$	Flight speed of drone type k
$v_{k}^{L},v_{k}^{E}$	Cruising speed of the k-th drone in loaded/empty state
$v^{b}$	Travel speed of the bus
$\sigma_{d},\sigma_{p}$	Constant service time per delivery/pickup at a customer location
$\sigma_{j}^{svc}$	Total service time at customer j
$\sigma_{h}$	Constant handling time at bus stops, including cargo (un)loading and battery replacement
$B_{sr}$	Arrival time of the r-th bus at stop s
$\left[E_{i},L_{i}\right]$	Time window at customer point i
$c_{vk},c_{fk},c_{rk}$	Unit flight cost, per-use cost, and escort (onboard) cost of drone type k
M	A sufficiently large constant
Variables	
$x_{ij}^{kmt}$	Binary variable: whether the m-th drone of type k flies from node i to node j during trip t
$y_{ss\text{'}}^{kmt}$	Binary variable: whether the m-th drone of type k travels with the bus from stop s to stop s' during trip t
$z_{is}^{kmtr}$	Binary variable: whether the m-th drone of type k boards the r-th bus from node i to stop s during trip t
$w_{j}^{kmt}$	Continuous variable: load of the m-th drone of type k when departing node j during trip t
$e_{j}^{kmt}$	Continuous variable: remaining flight range of the m-th drone of type k when arrival node j during trip t
$\eta_{ij}$	binary parameter, equal to 1 if arc (i,j) is operated in loaded state, and 0 otherwise
$\delta_{j}^{d}$	Binaryparameter: equals 1 if customer j requires a delivery service, and 0 otherwise
$\delta_{j}^{p}$	Binary parameter: equals 1 if customer j requires a pickup service, and 0 otherwise
$\tau_{j}^{kmt}$	Continuous variable: time when the m-th drone of type k when arrival node j during trip t
$u_{j}^{kmt}$	Continuous MTZ ordering variable (used for sub-tour elimination)

### Model assumptions

To highlight core research objectives and manage computational complexity, this study introduces the following assumptions without compromising essential contributions:

(1) Customer-related assumptions

- Customer locations and their respective delivery/pickup demands are predetermined and known.
- Each customer location is visited exactly once, fulfilling all associated delivery and pickup requests simultaneously.
- A constant service time is required at each customer location.

(2) Drone-related assumptions

- Time associated with drone takeoff and landing is considered negligible.
- Battery replacement and cargo loading/unloading occur at bus stops. A constant handling time is considered for these operations.
- Drones incur no additional costs while waiting in standby mode at bus stops.

(3) Bus-related assumptions

- Buses strictly adhere to fixed routes and schedules without delays or alterations.
- Bus dwell times at each stop are negligible.
- The initial station on each bus route also serves as the distribution depot.
- Each bus can simultaneously accommodate up to $H^{max}$ drones.

### Cost modeling of drone operations

Throughout the operational process, drones alternate between two distinct states: flying and hitchhiking (carried onboard buses). Each state impacts both model constraints and cost structures, requiring explicit formulation.

(1) Flight State

The drone’s flight state includes three types of movement: from bus stop to customer, between customers, and from customer back to a bus stop. The unit flight cost is denoted as $c_{vk}$, and a fixed per-use cost is denoted as *c*_*fk*_. Both components are incurred whenever a flying arc (*i,j*) is selected, so the total flight cost is obtained by summing over all chosen arcs. The total flight cost objective function *Z*_1_ is formulated as:


$$Z_{\text{1}}=\sum_{k\in K}\sum_{m\in M_{k}}\sum_{t\in T}[\sum_{i\in S}\sum_{j\in C}(c_{vk}d_{ij}^{d}+c_{fk})x_{ij}^{kmt}+\sum_{i\in C}\sum_{j\in C\backslash \left\{i\right\}}(c_{vk}d_{ij}^{d}+c_{fk})x_{ij}^{kmt}+\sum_{i\in C}\sum_{j\in S}(c_{vk}d_{ij}^{d}+c_{fk})x_{ij}^{kmt}]$$ (1)

(2) Hitchhiking state

To conserve battery power and facilitate cargo handling, drones utilize bus transport between stops. This hitchhiking incurs an escort cost denoted by *c*_*rk*_. The hitchhiking cost is also calculated arc by arc, i.e., for each bus segment (*s,s’*) traversed by a drone. The total drone hitchhiking cost objective function *Z*_2_ is expressed as:


$$Z_{\text{2}}=\sum_{s\in S}\sum_{s\text{'}\in S\backslash \left\{s\right\}}\sum_{k\in K}\sum_{m\in M_{k}}c_{rk}d_{ss\text{'}}^{b}y_{ss\text{'}}^{kmt}$$ (2)

### Optimization model formulation

This study aims to minimize the total operational cost of drones. The corresponding objective function and constraints are formulated as follows:


$$min\;F_{\text{2}}=Z_{\text{1}}+Z_{\text{2}}$$ (3)

s.t.


$$\begin{array}{c} \begin{array}{cc} \sum_{i\in N}\sum_{j\in C}d_{j}^{pkg}x_{ij}^{kmt}\leq Q_{k} & \end{array}\forall k\in K,\forall m\in M_{k},\forall t\in T \end{array}$$ (4)

$$\begin{array}{c} \begin{array}{cc} \sum_{i\in N}\sum_{j\in C}q_{j}^{ret}x_{ij}^{kmt}\leq Q_{k} & \end{array}\forall k\in K,\forall m\in M_{k},\forall t\in T \end{array}$$ (5)

$$\begin{array}{c} \begin{array}{cc} w_{j}^{kmt}\geq w_{i}^{kmt}+q_{j}^{ret}-d_{j}^{pkg}+M(x_{ij}^{kmt}-1) & \end{array}\forall k\in K,\forall m\in M_{k},\forall t\in T,\forall i\in N,\forall j\in C\backslash \left\{i\right\} \end{array}$$ (6)

$$\begin{array}{c} \begin{array}{cc} w_{j}^{kmt}\leq w_{i}^{kmt}+q_{j}^{ret}-d_{j}^{pkg}-M(x_{ij}^{kmt}-1) & \end{array}\forall k\in K,\forall m\in M_{k},\forall t\in T,\forall i\in N,\forall j\in C\backslash \left\{i\right\} \end{array}$$ (7)

$$\begin{array}{c} 0\leq w_{j}^{kmt}\leq Q_{k}\begin{array}{cc} & \forall j\in C, \end{array}\forall k\in K,\forall m\in M_{k},\forall t\in T \end{array}$$ (8)

$$\begin{array}{c} \sum_{t\in T}\sum_{i\in N}\sum_{k\in K}\sum_{m\in M_{k}}x_{ij}^{kmt}=1\begin{array}{cc} & \forall j\in C \end{array} \end{array}$$ (9)

$$\begin{array}{c} \sum_{j\in N}x_{oj}^{km1}=\sum_{i\in N}x_{ie}^{kmT_{max}}=1\begin{array}{cc} & \forall k\in K,\forall m\in M_{k} \end{array} \end{array}$$ (10)

$$\begin{array}{c} \sum_{s\in S}\sum_{j\in C}x_{sj}^{kmt}=1\begin{array}{cc} & \forall k\in K,\forall m\in M_{k} \end{array},\forall t\in T \end{array}$$ (11)

$$\begin{array}{c} \sum_{s\in S}\sum_{i\in C}x_{is}^{kmt}=1\begin{array}{cc} & \forall k\in K,\forall m\in M_{k} \end{array},\forall t\in T \end{array}$$ (12)

$$\begin{array}{c} \sum_{i\in C}x_{is}^{kmt}=\sum_{s\text{'}\in S}y_{ss\text{'}}^{kmt}\begin{array}{cc} & \forall k\in K,\forall m\in M_{k} \end{array},\forall t\in T \end{array}$$ (13)

$$\begin{array}{c} \sum_{j\in C}x_{s\text{'}j}^{km,t+1}=\sum_{s\text{'}\in S}y_{ss\text{'}}^{kmt}\begin{array}{cc} & \forall k\in K,\forall m\in M_{k} \end{array},\forall t\in T \end{array}$$ (14)

$$\begin{array}{c} \sum_{i\in N}x_{ij}^{kmt}=\sum_{l\in N}x_{jl}^{kmt}\begin{array}{cc} & \forall j\in C,\forall k\in K,\forall m\in M_{k} \end{array},\forall t\in T \end{array}$$ (15)

$$\begin{array}{c} \mu_{j}^{kmt}-\mu_{i}^{kmt}+1\leq (n+2)(1-x_{ij}^{kmt})\begin{array}{cc} & \forall k\in K,\forall m\in M_{k},\forall i\in N,\forall j\in N\backslash \left\{i\right\} \end{array},\forall t\in T \end{array}$$ (16)

$$\begin{array}{c} 1\leq \mu_{i}^{kmt}\leq n+2\begin{array}{cc} & \forall i\in N \end{array} \end{array}$$ (17)

$$\begin{array}{c} \sigma_{j}^{svc}=Max\left\{\delta_{j}^{d}\sigma_{d},\delta_{j}^{p}\sigma_{p}\right\}\begin{array}{cc} & \forall j\in C \end{array} \end{array}$$ (18)

$$\begin{array}{c} v_{k}^{d}=\eta_{ij}v_{k}^{L}+(1-\eta_{ij}v_{k}^{E})\begin{array}{cc} & \forall i,j\in N \end{array} \end{array}$$ (19)

$$\begin{array}{c} \tau_{j}^{kmt}\geq \tau_{i}^{kmt}+d_{ij}^{d}/v_{k}^{d}+\sigma_{j}^{svc}-M(1-x_{ij}^{kmt})\begin{array}{cc} & \forall k\in K,\forall m\in M_{k} \end{array},\forall i\in S,\forall j\in C,\forall t\in T \end{array}$$ (20)

$$\begin{array}{c} \tau_{j}^{kmt}\geq \tau_{i}^{kmt}+d_{ij}^{d}/v_{k}^{d}+\sigma_{j}^{svc}-M(1-x_{ij}^{kmt})\begin{array}{cc} & \forall k\in K,\forall m\in M_{k} \end{array},\forall i\in C,\forall j\in C/\left\{i\right\},\forall t\in T \end{array}$$ (21)

$$\begin{array}{c} \tau_{j}^{kmt}\geq \tau_{i}^{kmt}+d_{ij}^{d}/v_{k}^{d}-M(1-x_{ij}^{kmt})\begin{array}{cc} & \forall k\in K,\forall m\in M_{k} \end{array},\forall i\in C,\forall j\in S,\forall t\in T \end{array}$$ (22)

$$\begin{array}{c} \tau_{s}^{kmt}\leq B_{sr}+M(1-z_{is}^{kmtr})\begin{array}{cc} & \forall k\in K,\forall m\in M_{k} \end{array},\forall i\in C_{\text{1}},\forall s\in S,\forall t\in T,\forall r\in R_{s} \end{array}$$ (23)

$$\begin{array}{c} \sum_{r\in R_{s}}z_{is}^{kmtr}=x_{is}^{kmt}\begin{array}{cc} & \forall k\in K,\forall m\in M_{k} \end{array},\forall i\in C,\forall s\in S,\forall t\in T \end{array}$$ (24)

$$\begin{array}{c} \tau_{s\text{'}}^{kmt}\geq \tau_{s}^{kmt}+d_{ss\text{'}}^{b}/v^{b}+\sigma_{h}-M(2-z_{ij}^{kmtr}-y_{ss\text{'}}^{kmt})\begin{array}{cc} & \forall k\in K,\forall m\in M_{k} \end{array},\forall s,s\text{'}\in S,\forall r\in R_{s},\forall t\in T \end{array}$$ (25)

$$\begin{array}{c} \tau_{j}^{kmt}\geq B_{sr}+d_{sj}^{d}/v_{k}^{d}+\sigma_{j}^{svc}-M(2-z_{is}^{kmtr}-x_{sj}^{kmt})\begin{array}{cc} & \forall k\in K,\forall m\in M_{k} \end{array},\forall s\in S,\forall j\in C,\forall r\in R_{s},\forall t\in T \end{array}$$ (26)

$$\begin{array}{c} \sum_{i\in N}\sum_{j\in N\backslash \left\{i\right\}}d_{ij}^{d}x_{ij}^{kmt}\leq R_{k}\begin{array}{cc} & \forall k\in K,\forall m\in M_{k} \end{array},\forall t\in T \end{array}$$ (27)

$$\begin{array}{c} \sum_{k\in K}\sum_{m\in M_{k}}y_{ss\text{'}}^{kmt}\leq H^{max},\begin{array}{cc} & \forall s,s\text{'}\in S,\forall t\in T \end{array} \end{array}$$ (28)

$$\begin{array}{c} E_{i}\leq \tau_{i}^{kmt}\leq L_{i}\begin{array}{cc} & \forall k\in K,\forall m\in M_{k} \end{array},\forall i\in C,\forall t\in T \end{array}$$ (29)

$$x_{ij}^{kmt},y_{ss\text{'}}^{kmt},z_{is}^{kmtr},\delta_{j}^{d},\delta_{j}^{p},\eta_{ij}\in \left\{0,1\right\},\mu_{j}^{kmt},T_{j}^{kmt}\geq 0$$ (30)

(1) Drone capacity constraints (Eqs. (4)-(8))

These constraints impose the payload capacity limits of drones across different stages of a trip. Eq. (4) restricts the initial loading stage, ensuring that the number of parcels placed on the drone at its departure bus stop does not exceed its maximum payload. Eq. (5) applies to the pickup stage along the route, requiring that the cumulative amount of parcels collected while visiting customers also remains within the same payload limit. Eqs. (6)-(7) describe the capacity update stage at each customer node: after serving a customer, the remaining load is adjusted by the parcels picked up or delivered. The Big-M formulation ensures that this recursive update is only enforced when the corresponding arc (*i,j*) is selected, and otherwise relaxed. Finally, Eq. (8) bounds the residual payload within the interval between zero and the drone’s maximum capacity. Together, these constraints ensure that the drone’s payload limits are respected at departure, during customer visits, and throughout the entire mission.

(2) Routing and flow constraints (Eqs. (9)-(15))

These constraints ensure the logical integrity and connectivity of drone routes. Eq. (9) enforces service exclusivity, requiring that each customer node is visited exactly once by one drone, thus avoiding duplication or omission of service. Eq. (10) imposes the depot flow balance, ensuring that any drone dispatched from the distribution center must eventually return to it, thereby forming a closed and feasible route. Eqs. (11)-(12) guarantee the uniqueness of departure and landing bus stops, such that each drone trip selects exactly one take-off stop and one landing stop, preventing multiple invalid departures or arrivals. Eqs. (13)-(14) establish the connection between flight and hitchhiking states, ensuring consistent transitions between autonomous flying and bus-riding modes without logical breaks. Finally, Eq. (15) secures routing continuity, stipulating that after serving a customer, the drone must either proceed to another customer or return to a bus stop, thus avoiding dangling or incomplete paths. Collectively, these constraints guarantee that each drone route is spatially and logically connected, reflecting the operational requirements of real-world services.

(3) Sub-tour elimination constraints (Eqs. (16)-(17))

These constraints adopt the classical Miller–Tucker–Zemlin (MTZ) formulation to prevent the formation of isolated subtours in drone routing. By introducing auxiliary ordering variables, they enforce a consistent sequence of customer visits along each drone’s path, ensuring that all customers are integrated into a single connected route rather than fragmented cycles. In this way, the constraints maintain the structural feasibility of the routing solution and guarantee that drone tours correspond to realistic service operations.

(4) Drone-bus synchronization constraints (Eqs. (18)-(28))

A central challenge of the bus-drone collaborative delivery system is the temporal coordination between drone operations and bus schedules. Without careful synchronization, drones may arrive too late to re-board a bus, exceed their endurance during flight, or miss customer service windows, leading to infeasible or inefficient routes. To address this, Eqs. (18)-(28) collectively regulate the timing of drone movements under different operational modes and their alignment with bus services.

Eq. (18) defines the service time at each customer, determined by the type of demand (delivery, pickup, or both). Eq. (19) specifies the effective drone speed on each arc, distinguishing between loaded and empty states. Eqs. (20)-(22) govern the flying mode, ensuring that the drone’s arrival time at customers or bus stops is consistent with its flight distance and speed. Eqs. (23)-(24) impose the re-boarding condition, stipulating that if a drone intends to board a bus at stop s, it must return to that stop before the bus’s scheduled arrival. Eqs. (25)-(26) define the on-board (hitchhiking) mode, coordinating the timing of drone pickup, riding, and drop-off with the bus timetable. Eq. (27) enforces the flight endurance limit, constraining the total flying distance of a drone in each trip to its maximum range, while excluding segments where the drone is carried by a bus. Eq. (28) introduces the bus–drone interaction capacity, ensuring that the number of drones simultaneously carried by a bus does not exceed the carrying-capacity parameter $H^{max}$.

Together, these synchronization constraints constitute the core mechanism linking the drone routing problem to the bus timetable and capacity. By jointly regulating the timing of flights, re-boarding events, on-board movements, and carrying limits, they ensure that the multimodal system operates in a feasible, realistic, and well-coordinated manner, capturing the essence of bus–drone collaboration in rural logistics.

Finally, Eq. (29) enforces the time-window requirement at customer nodes, stipulating that parcel pickup must occur within the allowable service interval, thereby ensuring customer-oriented service quality. Eq. (30) specifies the domains of decision variables, defining the binary and continuous variables used in the formulation. Together, these constraints complement the operational logic by reflecting service quality requirements and maintaining the mathematical validity of the optimization model.

### Solution method

This study proposes a two-stage framework that combines a bus-stop–based clustering algorithm with an Improved Black Kite Algorithm (IBKA). The key idea is to exploit the natural hub role of bus stops to partition the large-scale bus–drone–customer network into several independent subregions; IBKA is then applied within each subregion, turning an exponential-size problem into a set of medium-scale subproblems that can be solved in parallel.

### Bus-stop–based nearest-neighbor clustering

A distinctive feature of our problem, compared with traditional PDVRP (Pickup and Delivery Vehicle Routing Problem), is that every physical bus stop is visited by both the forward- and reverse-direction buses. A drone may hitchhike in either direction to complete its mission. If the two directions were treated as separate nodes during clustering, the node set would double in size, artificially splitting a single flight into two, increasing take-offs/landings and inflating flight distance. To avoid this, we introduce a binary decision variable that records whether a drone boards the forward (0) or reverse (1) bus. During clustering, we fix each forward-direction stop as a cluster centroid; later, in the optimization stage, customer assignments can be remapped to the mirror stop when the binary variable equals 1. The clustering procedure is:

Step 1: Fixed centroids. Treat every forward-direction bus stop as a known cluster center.

Step 2: Feasibility filtering. Use the drone’s maximum flight range to filter feasible clusters.

Step 3: Nearest neighbor assignment. Assign each customer to the closest feasible bus stop.

The resulting clusters define independent PDVRP subproblems, each containing exactly one bus stop and its associated customers.

### BKA and IBKA

#### Black kite algorithm.

The BKA is a nature-inspired swarm intelligence optimization algorithm proposed by Wang et al [27]. It is modeled after the remarkable behaviors of the Elanus caeruleus. These behaviors include swift attacks on prey and adaptability to environmental conditions. Due to its simple structure, few parameters, and strong robustness, it has attracted widespread attention in the field of intelligent optimization. The detailed steps of the algorithm are as follows.

(1) Population Initialization

In BKA, the population initialization begins by generating a set of random solutions. The position of each black kite (BK) can be represented by the following matrix.


$$\begin{array}{c} X=\left[\begin{array}{c} @c@\begin{array}{c} X_{\text{1}}\\ \vdots \end{array}\\ X_{i}\\ \vdots \\ X_{pop} \end{array}\right]=\left[\begin{array}{ccccc} @ccccc@BK_{1,1} & \cdots & BK_{1,j} & \cdots & BK_{1,dim}\\ \vdots & \ddots & \vdots & \ddots & \vdots \\ BK_{i,1} & \cdots & BK_{i,j} & \cdots & BK_{i,dim}\\ \vdots & \ddots & \vdots & \ddots & \vdots \\ BK_{pop,1} & \cdots & BK_{pop,j} & \cdots & BK_{pop,dim} \end{array}\right] \end{array}$$ (29)

In the equation, *pop* denotes the population size in BKA, and *dim* represents the dimensionality of the decision variables.

In this study, each solution vector generated by Eq. (30) adopts a two-part chromosome structure. The first part encodes the service direction of each customer using a binary indicator: 0 denotes inbound service, and 1 denotes outbound service. The second part determines drone–customer assignments and service sequences: a continuous sub-vector is used to sort an auxiliary array containing both customer indices and separators for different drones, and each segment of the sorted array corresponds to the set and order of customers served by one drone. Through this decoding mechanism, any continuous vector can be consistently mapped into a discrete feasible solution, which then serves as the basis for routing and scheduling optimization.

Each element of ***X***_i_ is initialized randomly using the following formula.


$$X_{i}=BK_{lb}+rand(BK_{ub}-BK_{lb})$$ (30)

In the equation, *BK*_*lb*_ and *BK*_*ub*_ represent the lower and upper bounds of the *i*-th black kite in the *j*-th dimension, respectively, while *rand* is a random number within the interval [0,1].

Following initialization, the fitness of each individual in the population is evaluated. The individuals in ***X*** are then ranked based on their fitness values with lower fitness values indicating better performance.

(2) Attack Behavior

The black kite adopts two types of attack strategies: preparing to attack while circling in the air, and hovering while searching for prey. The mathematical model of these behaviors is as follows.


$$y_{t+1}^{i,j}=\left\{ \begin{array}{c} @l@y_{t}^{i,j}+n\times (1+sin(r))\times y_{t}^{i,j}\\ y_{t}^{i,j}+n\times (2r-1)\times y_{t}^{i,j} \end{array}\right.\begin{array}{c} p<r\\ else \end{array}$$ (31)

$$n=0.05\times e^{-2\times (t/T{)}^{\text{2}}}$$ (32)

In the equations, $y_{t}^{i,j}$ and $y_{t+1}^{i,j}$ represent the position of the *i*-th black kite in the *j*-th dimension at iteration steps *t* and *t* + 1, respectively. *T* is the total number of iterations, and *t* is the current iteration step. *p* is a constant, *p* = 0.9. *r* is a random number within the interval [0,1].

(3) Migration Behavior

Bird migration is typically guided by a leader, whose navigational ability is critical to the success of the group. In BKA, the following assumption is made: if the fitness of the current population is lower than that of a randomly chosen population, the leader gives up leadership and joins the migratory group, indicating it is not suitable to guide the population forward. Conversely, if the current population has higher fitness than the random one, it will guide the group toward the destination. The mathematical model of black kite migration behavior is presented below.


$$y_{t+1}^{i,j}=\left\{ \begin{array}{c} @l@y_{t}^{i,j}+C\left(0,1\right)\times (y_{t}^{i,j}-L_{t}^{j})\\ y_{t}^{i,j}+C\left(0,1\right)\times (L_{t}^{j}-m\times y_{t}^{i,j}) \end{array}\right.\begin{array}{c} F_{i}<F_{ri}\\ else \end{array}$$ (33)

In the equation, $L_{t}^{j}$ represents the leader kite’s position in dimension *j* at iteration *t*. *F*_*i*_ denotes the current position of the *i*-th black kite in dimension *j* at iteration *t*. *F*_*ri*_ denotes the fitness value of a randomly selected position in dimension *j* at iteration *t*. *C*(0,1) represents a one-dimensional Cauchy mutation.

The one-dimensional Cauchy distribution is a continuous probability distribution with two parameters. Its probability density function is given by:


$$\begin{array}{c} f\left(x,\delta ,\mu \right)=\frac{\text{1}}{\pi }\frac{\delta }{\delta^{\text{2}}+(x-\mu {)}^{\text{2}}}\begin{array}{cc} & -\infty <x<\infty \end{array} \end{array}$$ (34)

When δ = 1 and μ = 0, it becomes the standard form.


$$\begin{array}{c} f\left(x,\delta ,\mu \right)=\frac{\text{1}}{\pi }\frac{\text{1}}{1+x^{\text{2}}}\begin{array}{cc} & -\infty <x<\infty \end{array} \end{array}$$ (35)

### Improved Black Kite Algorithm

The BKA has been widely applied to continuous optimization thanks to its “leader-Cauchy jump” framework and its small number of control parameters. Recent comparative studies indicate that, when confronted with high-dimensional and highly constrained benchmarks or real engineering cases, BKA exhibits rapid diversity loss, premature convergence, and exploration–exploitation imbalance, so that solution quality and stability deteriorate markedly as problem size grows [28,29]. Given that our bus–drone coordination model is high-dimensional and includes several penalty constraints, directly using the standard BKA may lead to poor population coverage, mismatched search steps versus solution quality, and under-utilization of global-optimal directions. Accordingly, we modify four critical stages of the original BKA to create an IBKA.

(1) Opposition-Based Learning (OBL) for population initialization

In the original algorithm, Eq. (30) generates $X_{i}^{\left(0\right)}$ purely by uniform random sampling, which may over-populate certain regions while leaving their symmetric areas sparse, thereby reducing the first-generation search coverage. To enlarge the initial coverage, IBKA constructs an opposite individual ${\tilde{X}}_{i}^{\left(0\right)}$ for every $X_{i}^{\left(0\right)}$ (see Eq. 36) and then ranks the doubled population in ascending order of fitness, retaining only the best individuals. This procedure ensures that the initial solutions are evenly distributed over both ends of the variable range, giving the subsequent evolution a wider exploration radius.


$${\tilde{X}}_{i}^{\left(0\right)}=BK_{lb}+(BK_{ub}-BK_{lb})-(X_{i}^{\left(0\right)}-BK_{lb})$$ (36)

(2) Random shrinkage repair at the boundary

After the attack update (Eq. 31) or migration update (Eq. 33), if any component $y_{t+1}^{i,j}$ falls outside [*BK*_*lb,j*_*,BK*_*ub,j*_], the original algorithm clips it to the nearest bound, which often causes boundary crowding and weakens perturbation. Therefore, IBKA adopts a random shrinkage rule:


$$y_{t+1}^{i,j}=\left\{ \begin{array}{c} @c@BK_{lb,j}+0.5(BK_{ub,j}-BK_{lb,j})rand,\\ BK_{ub,j}-0.5(BK_{ub,j}-BK_{lb,j})rand,\\ y_{t+1}^{i,j} \end{array}\begin{array}{c} y_{t+1}^{i,j}<BK_{lb,j}\\ y_{t+1}^{i,j}>BK_{ub,j}\\ else \end{array}\right.$$ (37)

where *rand* is a random number uniformly drawn from [0,1]. This repair not only preserves feasibility but also re-injects 50% random variability into the violated dimension.

(3) Adaptive attack probability *p*_*i*_

In the original algorithm, Eq. (31) uses a fixed constant *p* = 0.9 to trigger two attack behaviors, forcing every individual to adopt the same transition amplitude. This setting cannot accommodate the different search-intensity requirements of solutions with varying quality. IBKA therefore defines *p*_*i*_ as a function of the individual fitness *F*_*i*_, see Eq. (38). This modification enables the step size to adapt to individual quality, thereby improving convergence speed and stability.


$$p_{i}=\left\{ \begin{array}{c} @c@p_{min},\\ p_{max}-\frac{p_{max}-p_{min}}{F_{max}-\overset{―}{F}}(F_{i}-\overset{―}{F}), \end{array}\right.\begin{array}{c} F_{i}<\overset{―}{F}\\ F_{i}\geq \overset{―}{F} \end{array}$$ (38)

In Eq. (38), $\overset{―}{F}$ and *F*_*max*_ denote the current mean and worst fitness, respectively. The constant *p* in Eq. (31) is then replaced by *p*_*i*_, while the original exponential-decay factor *n* is kept.

(4) Differential-evolution (DE) hybrid operator

The attack and migration phases of BKA do not explicitly exploit directional information from the current best individual *X*_*best*_. In the proposed IBKA, after each BKA update an additional DE round is applied to every individual, Eq. (39)-(41) show the mutation, crossover and dynamic *CR*(*t*) settings.


$$\begin{array}{c} V_{i}=X_{i}+0.5(X_{best}-X_{r}),\begin{array}{cc} & r\neq i \end{array} \end{array}$$ (39)

$$U_{ij}=\left\{ \begin{array}{c} @c@V_{ij},\\ X_{best,j}, \end{array}\right.\begin{array}{c} rand<CR\left(t\right)\\ else \end{array}$$ (40)

$$CR\left(t\right)=0.4+0.5\frac{t}{T}$$ (41)

If the fitness of *U*_*i*_ is better than that of *X*_*i*_, *U*_*i*_ replaces *X*_*i*_. The hybrid operator injects directional gradient information and, via the iteration-dependent crossover rate, enables a smooth shift from global exploration to local exploitation.

To provide a coherent view of the IBKA procedure, we summarize here the overall iterative process that integrates parameter updates, operator executions, and solution updates. At the beginning of each iteration, the step-size decay parameter is computed as Eq. (32), and the adaptive attack probability *p*_*i*_ is determined according to the relative fitness of each individual (Eq. 38). Each individual then sequentially undergoes three operators: the attack behavior (Eq. 31, with the small-step or large-step branch chosen by *p*_*i*_,), the migration behavior with Cauchy perturbation (Eq. 33–35), and the DE-based hybrid operator (Eq. 39–41, with a time-increasing crossover rate). Every candidate solution is subjected to half-span boundary repair (Eq. 37) and is immediately used to update the individual and, if better, the global best. This establishes a complete “parameter update–operator execution–solution update” loop, which provides the overall framework for the pseudocode presented in “Pseudo code of IBKA” subsection.

### Computational complexity

The efficiency of any algorithm is typically assessed by its time complexity [30]. For the basic BKA, generating *N* random kites and evaluating their objective values require *O*(*N* × *D*) and *O*(*N*) time, respectively. Each iteration then performs the attacking and migration behaviors, both of which involve D-dimensional vector operations for every individual, giving an iterative cost of *O*(*N* × *D*). Consequently, the overall time complexity of BKA is *O*(*T* × *N* × *D*).

IBKA retains these two vector updates and merely augments the algorithm with four lightweight procedures: (i) opposition-based learning generates a mirrored population in the same initial loop, leaving the *O*(*N* × *D*) order of the start-up phase unchanged; (ii) the self-adaptive computation of the attack probability *p*_*i*_ is a constant-time arithmetic step for each individual; (iii) the half-span rollback boundary repair scans a vector once and, when a component exceeds its bound, performs a single constant-time reassignment, thus not altering the order of complexity; and (iv) the best-rand differential-evolution operator adds one extra D‐dimensional update per kite in each generation.

Because all added operations are either *O*(1) or *O*(*D*) per individual, the per-iteration cost of IBKA remains *O*(*N* × *D*), yielding the same asymptotic time complexity *O*(*T* × *N* × *D*) as the original BKA while providing stronger exploration and exploitation capabilities.

### Space complexity

The space complexity of the IBKA algorithm is mainly determined by the storage of the population matrix. IBKA maintains the current population of size *N* × *D* together with a single temporary trial vector of size 1 × *D*; trial solutions are generated and evaluated individually and immediately compared with their parents, without storing the entire trial population. Hence, the total memory requirement is approximately (*N* + 1)×*D*. Auxiliary vectors used during mutation and crossover (of size *O*(*N*) or *O*(*D*)) are negligible compared with the population matrix, and the overall space complexity is *O*(*N* × *D*). Therefore, they do not increase the asymptotic memory demand relative to the original BKA.

### Pseudo code of IBKA

IBKA starts with opposition-based learning, generating mirrored individuals to widen the initial search range. During each iteration a fitness-driven, self-adaptive attack adjusts step size, while the standard migration is executed with Cauchy jumps and half-span boundary repair to preserve feasibility. Finally, a best–rand differential-evolution operator is applied, using a time-varying crossover rate to accelerate exploitation of the current global best. The full procedure is outlined in Table 3 of the Supporting information, which marks every modification relative to the original BKA.

**Table 3:** IBKA algorithm steps.

**Input**: pop (population size), T (max iterations), D (dim), BK_lower, BK_upper **Output:** X_best, F_best ----------------------------------------------------------------------- /* 1. Initialization with Opposition-Based Learning (Eq. 30 & 36) */ **For** i =1…pop **do** $X_{i}=BK_{lb}+rand(BK_{ub}-BK_{lb})$ → Eq. (30) ${\tilde{X}}_{i}^{\left(0\right)}=BK_{lb}+(BK_{ub}-BK_{lb})-(X_{i}^{\left(0\right)}-BK_{lb})$ → Eq. (36) Evaluate $F\left(X_{i}\right)$, $F\left({\tilde{X}}_{i}^{\left(0\right)}\right)$ and add to pool **end for** pool ← $\left\{X_{i},{\tilde{X}}_{i}\right\}$; evaluate F(pool); sort pool ascending by F X ← first pop individuals of pool; $F_{i}$ ← $F\left(X_{i}\right)$ $X_{best}\leftarrow X_{\text{1}}$; $F_{best}\leftarrow F_{\text{1}}$ ----------------------------------------------------------------------- /* 2. Main evolutionary loop */ **For** t = 1…T **do** // terminate when t reaches the maximum iteration T Compute $F_{avg}$, $F_{max}$; Leader ← current best **For** i =1…pop **do** /* 2.1 Self-adaptive attacking behavior */ $p_{i}$ ← Eq. (38) using $F_{i}$, $F_{avg}$, $F_{max}$ If $p_{i}$ < rand then $Y_{i}$ ← Eq. (31), first branch/* small step */ Else $Y_{i}$ ← Eq. (31), second branch/* large step */ End if If $Y_{i}$ is out of bounds then repair by Eq. (37) End if/* half-span rollback */ If $F\left(Y_{i}\right)<F_{i}$ then $X_{i}\leftarrow Y_{i}$, $F_{i}\leftarrow F\left(Y_{i}\right)$ End if /* 2.2 Migration behavior with Cauchy flight */ ${Y_{i}}^{\prime }$ ← Eq. (33) using C(0,1) defined by Eq. (34)–(35) If ${Y_{i}}^{\prime }$ is out of bounds then repair by Eq. (37) End if/* half-span rollback */ If $F\left({Y^{\prime }}_{i}\right)<F_{i}$ then $X_{i}\leftarrow {Y_{i}}^{\prime }$, $F_{i}\leftarrow F\left({Y_{i}}^{\prime }\right)$ End if /* 2.3 DE–based hybrid operator */ Choose r≠i uniformly $V_{i}=X_{i}+0.5(X_{best}-X_{r})$ → Eq. (39) $U_{i}$ ← binomial-crossover($V_{i}$, $X_{best}$, CR(t)) → Eq. (40) with CR(t)=0.4+0.5t/T → Eq. (41) If $U_{i}$ is out of bounds then repair by Eq. (37) End if If $F\left(U_{i}\right)<F_{i}$ then $X_{i}\leftarrow U_{i}$, $F_{i}\leftarrow F\left(U_{i}\right)$ End if If $F_{i}<F_{best}$ then $X_{best}\leftarrow X_{i}$, $F_{best}\leftarrow F_{i}$ End if **End for** /* end population loop */ **End for** /* stopping criterion satisfied: t = T */ **Return** X_best, F_best

### Numerical results

#### Hypothetical instances.

Because the existing literature offers no unified benchmark for the “bus-drone collaborative pickup-and-delivery with time windows” problem, this study scientifically and systematically assesses the proposed model and the IBKA by adapting three representative instances—LC101, LR101, and LRC101—from the Li & Lim extension of the Solomon dataset. Each instance is rescaled to customer sets of 10, 25, 50, 75, and 100 nodes, resulting in 15 test groups. In LC101, customers are cluster-distributed to test the scheduling efficiency in high-density areas; in LR101, customers are randomly scattered, simulating evenly distributed demand along the route; in LRC101, customers follow a mixed (random + clustered) pattern, combining dense cores with outlying points to gauge overall adaptability.

In every instance, the bus route is a polyline that passes through ten stops: (0,0) → (10,10) → (20,20) → … → (90,90) → (100,100), with (0,0) serving both as the origin terminal and as the depot. Each stop can serve as a drone take-off, landing, and replenishment node, allowing a uniform route framework for systematically comparing the impact of customer size and spatial distribution on solution performance.

### Parameter Settings and Experimental Environment

Customer coordinates in the Solomon instances are located within a 100 × 100 planar grid. To match real-world geography and operating time, one distance unit in the Solomon files is interpreted as 0.1 km and one time unit as 0.5 min; the time origin is set to 06:00. Drone performance parameters follow SF UAV specifications and related literature [31,32]. Operational cost, maximum range, speed, and payload for the three drone types are listed in Table 4. The heterogeneous fleet is fixed at two type-A drones and one drone each of types B and C, reflecting the limited bus cargo space and ensuring that range/payload differences across drone types are represented. Service operations incorporate constant processing times: 2 minutes for each delivery, 10 minutes for each pickup, and 10 minutes at bus stops for cargo handling and battery replacement. In rural passenger transport, terminal stops are often in remote villages where parking or maintenance facilities are unavailable. Buses therefore operate on a “round-trip” pattern: departing the depot, reaching the far end of the route, and immediately returning to the origin to finish one outbound-and-return cycle. Given that rural bus services are much less frequent than their urban counterparts, the headway is set to 120 min. Operating hours are 06:00–18:00, and buses travel at a constant speed of 25 km/h. The bus–drone interaction capacity is set to $H^{max}=3$ in all experiments.

**Table 4:** Drone Performance Parameters.

Type	Delivery Cost (¥/km)	Per-Use Cost (¥/mission)	Hitchhiking Cost (¥/ride)	Payload (kg)	Range (km)	Loaded Cruise Speed (km/h)	Empty Cruise Speed (km/h)
A	0.15	2	5	20	60	70	90
B	0.2	3	5	30	30	70	90
C	0.25	6	5	60	20	40	60

To benchmark the IBKA, three comparison methods were configured as follows:

- Gurobi 12.0.2. The complete MILP model was submitted directly to the solver. The 10-customer instance was solved to proven optimality with no time limit, whereas the 25-, 50-, 75- and 100-customer instances were each limited to 1 800 s of wall-clock time.
- Standard Genetic Algorithm (GA). Population size 200, generations 200, crossover probability 0.90, mutation probability 0.03, roulette-wheel selection, and elitist preservation of the best individual.
- Eel and Grouper Optimizer (EGO). Configured according to Mohammadzadeh and Mirjalili (2024), where the Eel and Grouper Optimizer was originally proposed [33]. Population size and number of iterations are kept identical to the GA for consistency, and all other control parameters follow the recommended defaults in the original paper.
- Standard BKA. Attack probability fixed at 0.90 and the step-size decay constant in Eq. (32) set to 0.05, both following Wang et al. [27]; population size and number of iterations identical to the GA; all other parameter settings follow the “Black Kite Algorithm” subsection.
- The proposed IBKA augments the BKA with opposition-based initialization, adaptive attack probability, random boundary shrinkage, and a DE hybrid operator (see the “Improved Black Kite Algorithm” subsection for details).

Each algorithm—Gurobi, GA, EGO, BKA, and IBKA—was executed ten independent runs on identical hardware (AMD Ryzen 5 5500 @ 3.59 GHz, 16 GB RAM) under Python 3.11; Gurobi was accessed through its Python API. For every run we recorded the best objective values and computed the coefficient of variation (CV)—the ratio of the standard deviation to the mean—to quantify solution stability. Detailed results are summarized in Table 5.

**Table 5:** Results from hypothetical instances.

Size	Instance	GA			EGO			BKA			IBKA		
		BestV	CV	Time	BestV	CV	Time	BestV	CV	Time	BestV	CV	Time
10	LC101	128.99	0.01	42.76	128.95	0.01	123.58	129.02	0.01	126.61	128.95	0.00	165.90
	LR101	140.24	0.01	40.46	140.24	0.00	107.04	140.24	0.00	117.58	140.24	0.00	149.35
	LRC101	128.52	0.02	40.08	128.52	0.02	117.08	128.54	0.03	122.64	128.51	0.00	161.11
25	LC101	153.34	0.49	63.30	154.70	2.11	174.24	155.87	1.78	174.21	152.38	0.16	201.75
	LR101	144.74	0.26	58.43	144.37	0.17	163.50	144.78	0.91	161.99	142.19	0.05	197.53
	LRC101	159.59	0.79	58.37	163.05	23.79	188.53	172.30	27.03	175.52	158.85	0.78	218.78
50	LC101	159.05	16.05	75.73	173.18	31.74	229.68	286.50	14.45	204.79	155.05	15.51	272.96
	LR101	154.13	15.67	79.18	170.85	22.77	233.48	168.43	17.79	208.09	146.70	11.42	264.53
	LRC101	253.67	15.87	82.83	307.94	30.56	240.12	302.67	17.45	213.73	170.24	10.99	287.05
75	LC101	280.70	9.03	102.81	325.02	24.41	293.67	302.87	22.57	245.16	266.03	11.38	352.10
	LR101	309.82	11.97	114.39	466.49	22.57	296.79	–	–	239.17	307.01	13.58	352.34
	LRC101	281.34	13.52	111.24	531.10	23.57	302.38	–	–	242.67	266.01	11.31	352.85
100	LC101	500.34	15.89	133.70	–	–	354.16	–	–	267.92	404.66	13.85	400.78
	LR101	377.09	11.58	133.47	–	–	347.08	–	–	265.77	314.66	9.26	396.56
	LRC101	425.57	10.78	129.91	–	–	346.40	–	–	264.75	388.99	11.57	397.37

### Algorithm performance analysis

For the 10-customer cases, Gurobi is able to reach the global optimum (128.95 for LC101, 140.24 for LR101, and 128.51 for LRC101). As the problem size increases, however, its performance deteriorates sharply—within the 1 800 s time limit it only returns a sub-optimal solution for LR101 with 25 customers (gap 10.59%), and fails to yield any feasible solution for the remaining larger instances. Notably, IBKA also achieves the exact optimal solutions in the 10-customer cases, which confirms its correctness and shows that the algorithm can reach global optima when the problem size is tractable.

In terms of solution quality, the ranking is clear: IBKA > GA > EGO ≈ BKA, and this superiority becomes more pronounced as the instance size grows. Regarding solution stability, IBKA exhibits the strongest robustness across all problem sizes, with CV values generally within 0–15%. GA ranks second in stability, though it fails twice on LC101−100 and three times on LRC101−100. By contrast, the standard BKA fails to obtain any feasible solution in all ten runs for LR101−75, LRC101−75, and the three 100-customer instances. EGO also fails in all ten runs for the three 100-customer cases. IBKA, on the other hand, misses only once on LC101−100 (the reported CV excludes that single invalid run).

**Fig 2:**
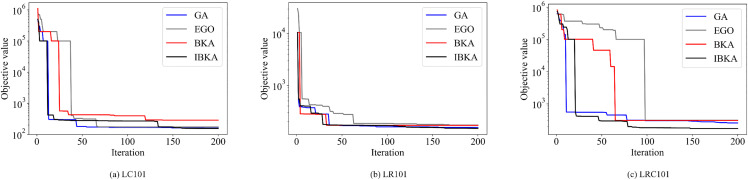
Convergence process of GA, EGO, BKA, and IBKA.

As for convergence speed, Fig 2 compares GA, EGO, BKA, and IBKA on the 50-customer instances LC101, LR101, and LRC101. The figure clearly shows that IBKA rapidly approaches near-optimal solutions in the early stages and maintains the lead throughout iterations, converging faster than GA and BKA and demonstrating superior global search ability and efficiency.

We conducted Wilcoxon signed-rank tests using the best objective value (BestV) of each instance as the performance indicator, and formed paired comparisons between IBKA and each competing algorithm. As reported in Table 6, all tests return a Wilcoxon statistic of zero with p-values below 0.01. This shows that, across all feasible instances IBKA never performs worse than GA, EGO, or BKA, and is strictly better in several cases, providing strong statistical evidence that its superiority is systematic rather than incidental.

**Table 6:** Wilcoxon signed-rank test results.

Comparison	N	W (statistic)	p-value
IBKA vs GA	15	0.00	0.00098
IBKA vs EGO	12	0.00	0.00506
IBKA vs BKA	10	0.00	0.00769

### Ablation study

To further evaluate the contribution of each enhancement strategy, we conducted an ablation study on the four core mechanisms of IBKA, namely opposition-based learning (O), adaptive attack probability (A), stochastic boundary shrinkage (S), and DE-style mutation (D). The results are summarized in Table 7.As shown, DE-style mutation (D) plays a decisive role, delivering substantial improvements across all three benchmark instances and achieving performance levels close to IBKA even when used alone. In contrast, adaptive attack probability (A) and opposition-based learning (O) provide only limited improvements in certain cases, indicating that their function is mainly supportive. Notably, stochastic boundary shrinkage (S) deteriorates solution quality when applied in isolation, suggesting that it must be combined with other mechanisms to exert a positive effect. From the perspective of combined effects, O + S or O + S + A does not yield significant improvements over the baseline BKA, confirming that partial mechanisms are insufficient. By comparison, IBKA, integrating all four mechanisms, consistently achieves the best performance with the lowest variation (CV%), demonstrating that IBKA’s superiority primarily stems from the leading contribution of D, while O and A enhance convergence stability and robustness when working in synergy with D.

**Table 7:** Ablation study results of algorithm variants.

Algorithm	LC101		LR101		LRC101	
	BestV	CV (%)	BestV	CV (%)	BestV	CV (%)
BKA	286.50	14.45	168.43	17.79	302.67	17.45
BKA + D	155.60	11.02	147.95	16.57	177.56	11.15
BKA + A	173.21	16.45	168.45	20.57	302.19	15.97
BKA + S	287.94	14.05	181.62	15.64	442.37	18.84
BKA + O	180.65	11.19	168.63	16.37	289.12	17.67
BKA + O + S	280.42	15.32	167.54	12.37	302.01	10.75
BKA + O + S + A	280.37	15.26	167.57	18.67	302.64	17.14
IBKA	155.05	15.51	146.70	11.42	170.24	10.99

**Fig 3:**
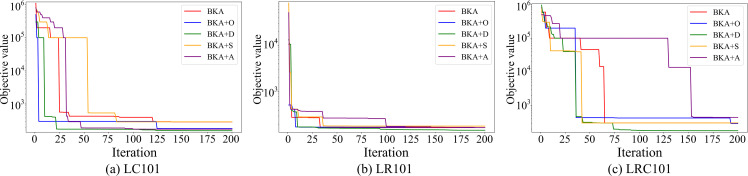
Convergence curves of algorithm variants in the ablation study.

To provide further insight into how the mechanisms affect the search process, we plotted the convergence curves of BKA, BKA + D, BKA + A, BKA + S, and BKA + O on three representative instances (see Fig 3). The curves reveal distinct patterns: opposition-based learning (O) accelerates convergence in the early stages by enriching population diversity; adaptive attack probability (A) sustains progress in the middle and later stages, preventing premature stagnation; stochastic boundary shrinkage (S), when applied alone, shrinks the search space too aggressively, leading to insufficient exploration and poor solution quality; and DE-style mutation (D) demonstrates the most favorable performance, showing smooth and stable convergence while effectively approaching the global optimum. These observations are consistent with the numerical results in Table 7, confirming that each mechanism plays a specific role but remains limited in isolation, whereas their integration in IBKA achieves a balanced synergy between early exploration and later exploitation, resulting in superior solution quality and robustness.

### Sensitivity analysis

#### Algorithm-related parameter sensitivity.

To evaluate the robustness of IBKA against parameter settings, we conducted sensitivity tests on four key parameters: the adaptive attack probability range *P*, the population size *pop*, the maximum iteration number *T*, and the DE parameter settings (mutation factor *F* and crossover rate *CR*). For each parameter, one factor was varied while others were fixed at their default values.

(1) Population size. As shown in Table 8, when the population size is too small (e.g., 50 or 100), the best solution deteriorates substantially. Increasing the population size to 150 or higher greatly improves solution quality, though at the expense of computation time. Beyond 200, further improvements are negligible relative to the cost increase. Thus, a moderate population size (150–200) offers the best trade-off between efficiency and effectiveness.
(2) Iteration number. Table 9 indicates that insufficient iterations (≤100) result in premature termination and inferior solutions. Once the iteration number reaches 200, IBKA consistently converges to high-quality solutions, and larger values (≥300) do not yield significant gains but incur higher computation time.
(3) Attack probability range. The results in Table 10 show that different settings of P lead to nearly identical best solutions across all instances, with only minor variations in the coefficient of variation. This insensitivity is largely attributable to the adaptive attack probability mechanism introduced in IBKA, which dynamically adjusts the probability during the search process. As a result, the algorithm remains stable without requiring fine-tuned bounds for *p*.
(4) DE parameter settings. A sensitivity analysis was conducted on the mutation factor *F* and crossover rate *CR* of the DE operator, as these two parameters directly control the search step size and information exchange. The tested ranges (*F* = 0.3–0.9; *CR* varying dynamically from different lower to upper bounds, e.g., [0.4,0.9]) were selected based on common practice in the DE literature. The results (Table 11) show that an excessively small *F* = 0.3 leads to poor solutions, while values between 0.5 and 0.9 remain stable. For *CR*, wider ranges such as [0.4,0.9] perform better than narrower intervals, ensuring balanced exploration and exploitation. These findings confirm that our initial settings (*F* = 0.5, *CR*∈[0.4,0.9]) are robust and appropriate across instances.

**Table 8:** Sensitivity analysis on the population size.

pop	LC101			LR101			LRC101		
	BestV	CV (%)	Time(s)	BestV	CV (%)	Time(s)	BestV	CV (%)	Time(s)
50	162.92	26.75	73.71	148.27	24.8	72.2	251.53	14.07	76.25
100	170.29	24.72	161.73	148.51	25.8	145.7	262.06	13.78	158.01
150	155.37	27.04	223.25	148.26	27.1	224.6	170.39	23.67	236.8
200	155.05	15.51	272.96	146.70	11.42	264.53	170.24	10.99	287.05
250	155.58	22.32	387.31	146.50	25.8	349.1	170.10	14.07	386.5
300	154.79	24.97	462.02	146.10	22.2	402.9	170.36	13.03	456.5

**Table 9:** Sensitivity analysis on the maximum iteration number.

T	LC101			LR101			LRC101		
**BestV**	**CV (%)**	**Time(s)**	**BestV**	**CV (%)**	**Time(s)**	**BestV**	**CV (%)**	**Time(s)**
50	165.16	28.20	73.37	230.96	12.80	69.61	173.12	28.60	74.14
100	155.98	26.00	147.00	149.02	21.20	140.08	171.18	21.30	150.17
150	156.25	28.30	224.29	149.68	22.50	210.63	170.33	17.90	226.63
200	155.05	15.51	272.96	146.70	11.42	264.53	170.24	10.99	287.05
300	155.01	24.50	434.01	146.68	24.70	406.00	170.22	10.58	428.08
400	155.02	26.80	560.92	146.06	19.50	541.44	170.20	10.37	585.95

**Table 10:** Sensitivity analysis on the adaptive attack probability range.

P	LC101		LR101		LRC101	
	BestV	CV (%)	BestV	CV (%)	BestV	CV (%)
[0.45, 0.85]	155.17	25.25	148.48	14.64	170.68	15.67
[0.45, 0.90]	155.28	25.03	150.56	15.53	169.91	14.40
[0.45, 0.95]	155.02	31.46	146.36	20.48	171.22	16.34
[0.60, 0.85]	155.63	22.61	147.15	22.72	170.16	18.75
[0.60, 0.90]	155.05	15.51	146.70	11.42	170.24	10.99
[0.60, 0.95]	155.74	27.87	148.37	23.59	170.56	10.98
[0.70, 0.85]	155.32	22.19	147.25	22.85	170.10	11.27
[0.70, 0.90]	154.98	22.97	146.17	22.02	171.54	15.64
[0.70, 0.95]	154.98	22.21	147.98	23.37	171.29	16.28

**Table 11:** Sensitivity analysis on DE parameter settings.

F/CR(t)	LC101		LR101		LRC101	
	BestV	CV (%)	BestV	CV (%)	BestV	CV (%)
F=0.3	161.92	23.52	147.63	15.79	254.10	18.68
F=0.5	155.05	15.51	146.70	11.42	170.24	10.99
F=0.7	155.19	16.29	147.69	13.48	168.96	15.79
F=0.9	155.65	15.23	148.18	11.45	169.27	13.78
*CR* (*t*) ∈ [0.4, 0.7]	159.15	21.56	151.38	13.67	277.60	19.67
*CR* (*t*) ∈ [0.4, 0.9]	155.05	15.51	146.70	11.42	170.24	10.99
*CR* (*t*) ∈ [0.5, 0.7]	158.00	15.47	152.76	12.57	172.51	11.97
*CR* (*t*) ∈ [0.6, 0.9]	154.76	14.57	147.51	13.78	178.12	15.78

Overall, the sensitivity analysis demonstrates that IBKA remains robust across a wide range of parameter settings, with only population size and iteration number requiring moderate tuning, while the adaptive attack probability and DE parameters perform reliably within commonly used ranges.

### Model-related parameter sensitivity

This study selects drone range, drone payload, and bus departure frequency as key parameters for sensitivity analysis. The drone’s range determines its effective flight radius and thus the spatial coverage of the delivery network; analyzing this parameter helps accurately assess system performance. Likewise, payload capacity dictates the load carried per sortie and directly affects operating cost and service level; examining its variation is vital for optimizing operational strategy. In addition, the bus departure interval plays a key role in service quality and cost reduction; setting an appropriate headway can markedly improve delivery efficiency, especially under varying customer densities.

**Fig 4:**
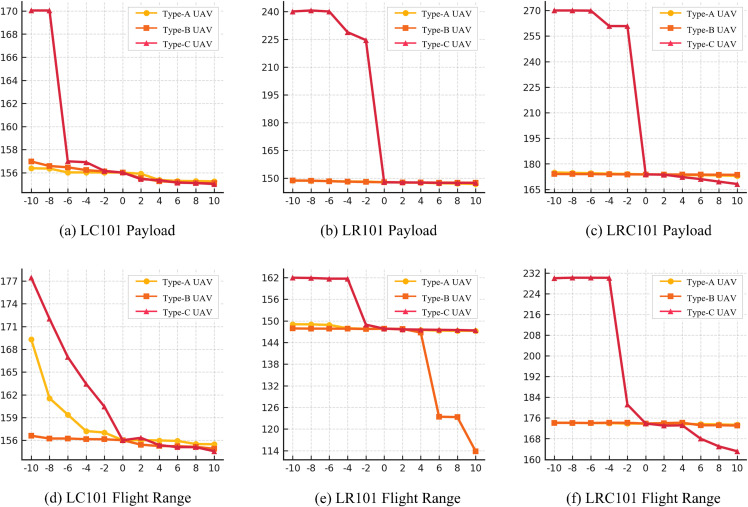
Results of Drone Payload and Range Sensitivity Analysis.

The 50-customer instances LC101, LR101, and LRC101 are used as test beds, because their different spatial patterns emulate the complex distribution of rural demand points. During testing, the range of type-A drones varies from 50 km to 70 km and the payload from 10 kg to 30 kg; for type B, the ranges are 20 km to 40 km and the payloads 20 kg to 40 kg; for type C, 10 km to 30 km and 50 kg to 70 kg. All range and payload values change in 2-unit increments. Bus departure intervals are examined from 0.5 h upward, increasing in 0.5-h steps to 9 h. The corresponding results are presented in Fig 4 and Table 12.

**Table 12:** Results of bus departure-interval sensitivity analysis.

LC101		LR101		LRC101	
Departure Interval (h)	BestV	Departure Interval (h)	BestV	Departure Interval (h)	BestV
1	155.84	1	147.25	1	173.56
2	156.02	2	147.83	2	173.82
3	156.61	3	148.51	3	173.56
4	376.87	4	148.36	4	425.87
5	379.99	5	148.12	5	426.43
6	379.83	6	148.79	6	425.67
7	--	7	243.54	7	--
8	--	8	243.78	8	--

Fig 4 indicates that, in the 50-customer scenarios, type-C drones—characterized by high payload and short range—produce the most pronounced cost reductions when either range or payload is increased from its baseline. As range or payload grows, a type-C drone can serve more customers per sortie, enhancing task consolidation, shortening empty repositioning legs, and thus lowering total operating cost. Nevertheless, the marginal benefit tapers off once range or payload exceeds intermediate values. By contrast, type-A drones (low payload, long range) display a noticeable cost decline only in the LC101 payload test, with negligible change elsewhere. Type-B drones (medium payload and range) achieve modest savings only in the later stages of the LR101 payload experiment, suggesting that types A and B are more sensitive to specific task structures and customer spatial patterns.

Table 12 reveals a clear step-wise surge in total operating cost as the bus departure interval lengthens. Sparse schedules sharply reduce drone boarding opportunities and routing flexibility, which in turn lowers overall efficiency, raises delivery cost, and—at extreme headways—can even render certain tasks infeasible, jeopardizing service continuity.

Overall, the sensitivity study reveals that high-payload drones are more sensitive to parameter changes, and tuning their range and payload has a larger direct impact on total cost and efficiency. Therefore, practical deployments should prioritize parameter setting and fleet composition for high-payload drones to balance performance and cost.

### Case study

This study uses the rural bus route from Xunyang City to Tongqianguan Town, Shaanxi Province, as a case to apply and analyze the proposed model and algorithm. In recent years, government agencies and courier companies in Xunyang have actively explored an integrated “passenger–freight–post” rural logistics system; the Xunyang–Tongqianguan route is a flagship demonstration line for this initiative. At present, parcels are mainly carried on buses and dropped at village service stations along the route, with an average of roughly 900 items trans-shipped per month. Although the scheme shows promise, the network extends only to township-level service points; true door-to-door delivery is still lacking, leaving considerable room to improve service efficiency and response time.

**Fig 5:**
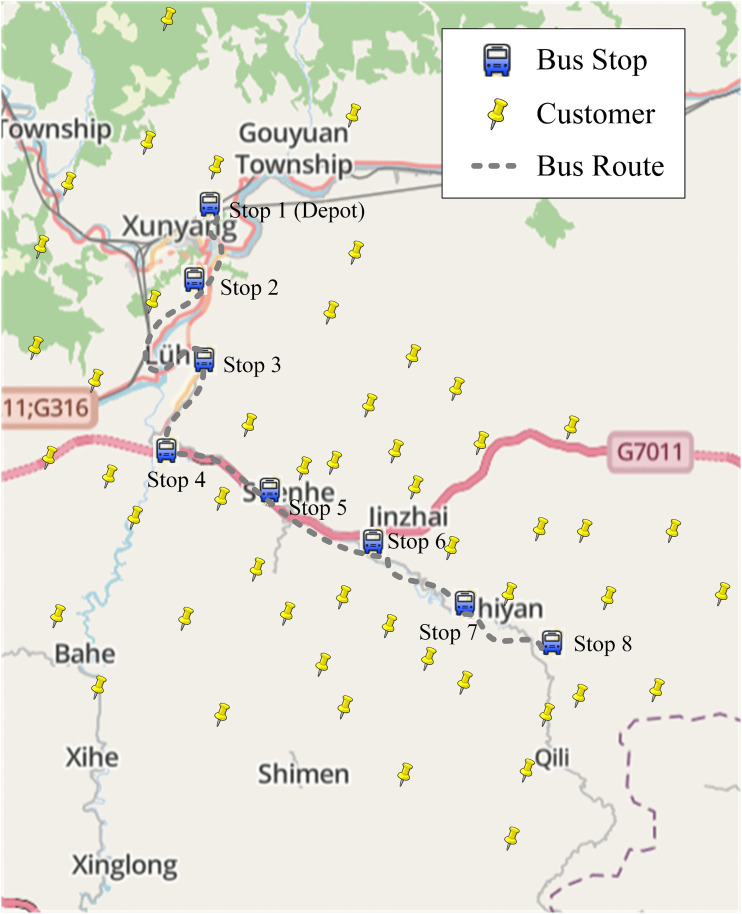
Case study. **Basemap data:** OpenStreetMap contributors, made available under the Open Database License (ODbL) v1.0 (https://www.openstreetmap.org/copyright).

The route spans 44.8 km, operates eight round-trips per day between 06:00 and 18:00, passes four townships, and serves more than ten villages and 70 residential clusters. Eight fixed bus stops are used for passenger boarding and freight transfer (Fig 5). Deliveries in the study area are typically made only to administrative-village pickup points rather than to individual customer addresses, so geocoded last-mile customer locations are not available. To approximate the local service setting, we randomly generate 50 customer nodes within a 15 km-wide corridor on both sides of the route to represent rural end users or merchants. Demand data are taken from the 2024 Xunyang township logistics audit ledger to ensure accuracy and reliability. Drone and bus parameters are consistent with those used in the numerical experiments described in the “Parameter Settings and Experimental Environment” subsection.

To further evaluate the proposed bus-assisted heterogeneous-drone mode, two benchmark schemes are considered: (i) a conventional truck-only operation and (ii) a bus-assisted homogeneous scheme that employs only type-C drones. In the truck mode, a conventional lorry with a 500 kg payload performs all deliveries. The truck is assumed to have an average purchase price of ¥100,000, depreciated over ten years with 270 operational days per year, which yields a fixed daily cost of roughly ¥37. Based on a bottom-up estimation, the fuel cost of a light-duty truck in China is approximately ¥0.68 per km (assuming 10 liters per 100 km at the current diesel price of ¥6.76 per liter, updated September 22, 2025). Labor cost is estimated at ¥0.85 per km, calculated from the 2024 national average annual wage in the postal and express delivery sector (¥67,973) and an annual mileage of 80,000 km. Taken together, these estimates correspond to a total operating cost of approximately ¥1.53 per km.

Table 13 compares the total costs. The bus-assisted heterogeneous-drone scheme incurs only ¥683.58, substantially below the ¥1088.11 required by the truck-only mode, underscoring its economic advantage. The homogeneous scheme, limited to type-C drones, cannot produce a feasible solution when the drone range is 20 km; even after extending the range to 40 km (with all other parameters unchanged), its cost still exceeds that of the heterogeneous fleet.

**Table 13:** Comparison of Total Cost for Different Delivery Modes.

Delivery Mode	Bus + Heterogeneous Drones	Bus + Type-C Drones (20 km range)	Bus + Type-C Drones (40 km range)	Truck Delivery
Total Cost (¥)	683.5879	--	1034.46	1088.11

**Fig 6:**
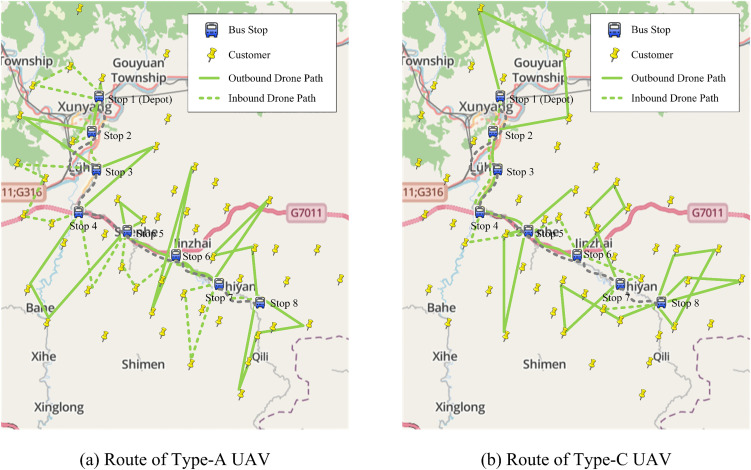
Case study solution. **Basemap data:** OpenStreetMap contributors, made available under the Open Database License (ODbL) v1.0 (https://www.openstreetmap.org/copyright).

Fig 6 illustrates the optimal schedule produced by the bus-assisted heterogeneous-drone scheme in the Xunyang-Tongqianguan case. All pickup-and-delivery tasks are covered by one type-A drone and one type-C drone. The type-A drone, with its long range and lighter payload, serves customers located farther from the bus corridor, whereas the type-C drone—with its higher payload but shorter range—handles larger single-node orders and dense clusters near successive bus stops. Their complementary strengths in flight radius and carrying capacity enable efficient resource allocation and an overall service configuration that minimizes total operating cost.

These results confirm the practicality of the proposed framework in complex rural settings and demonstrate its economic efficiency in the examined case. The heterogeneous drone fleet provides robust cost savings relative to the benchmark configurations, highlighting the value of capability complementarity under dispersed demand and limited rural transit supply.

### Managerial Insights

This chapter translates the sensitivity analysis and case-study results into actionable managerial insights for rural bus-assisted drone operations, focusing on service frequency, fleet composition, and capability specification. The recommendations are grounded in the numerical evidence presented in this study.

First, service frequency is a regime-defining factor. The sensitivity analysis shows that bus headway is a primary driver of both cost performance and operational viability in bus-assisted drone systems. As summarized in Table 12, sufficiently frequent timetable connectivity supports stable performance, whereas longer headways can shift the system into a degraded regime characterized by sharp cost escalation and, in extreme cases, infeasibility. From a managerial perspective, ensuring adequate bidirectional connection opportunities should be treated as a prerequisite for deployment, and improving timetable connectivity may yield greater benefits than marginal drone capability upgrades when transit supply is sparse.

Second, high-payload drones matter most. The sensitivity analysis indicates that system performance is most responsive to changes in payload and range for the high-payload drone type, whereas improving the long-range, low-payload type tends to yield smaller marginal effects in most tested settings, with diminishing returns as capability continues to increase (Fig. 4). Operationally, this suggests prioritizing a high-payload segment whose capability is aligned with the dominant demand profile, stop spacing, and connection opportunities, while avoiding over-specification once a critical capability level is achieved in order to reduce unnecessary operational burden.

Third, heterogeneous fleets are more advantageous in rural settings. The case-study results show that when customers are more dispersed and parcel weights are more diverse, a heterogeneous fleet can more readily exploit complementarities—using long-range drones to reach remote customers and high-payload drones to serve heavier orders—to improve both accessibility and operational efficiency, leading to more robust cost performance (Table 13). By contrast, homogeneous fleets are often more suitable for typical urban distribution contexts, where demand density is higher and customers are more concentrated, and operations can be more easily organized by service area or weight class; in such cases, a single drone type can cover most service needs while simplifying fleet management and maintenance.

Overall, these findings suggest a clear design logic for rural bus-assisted drone deployment. Service frequency and bidirectional timetable connectivity should be assessed first to ensure that the system operates in a viable and economically stable regime. Within that operational envelope, fleet composition and capability specification should be tailored to local demand characteristics by prioritizing high-payload capability and adopting heterogeneous fleets when dispersion and weight heterogeneity are pronounced. Together, these insights provide practical guidance for selecting service configurations and fleet strategies to achieve reliable, cost-effective rural pickup-and-delivery operations.

## Conclusion

This study advances the bus–drone collaboration literature by addressing a rural operating context that is underrepresented in prior work, which has largely focused on high-frequency urban transit networks, one-way last-mile delivery, and homogeneous drone fleets. We propose a rural collaborative pickup-and-delivery framework that integrates fixed-route buses with heterogeneous drones, explicitly modeling bidirectional logistics flows and incorporating two-way fixed bus timetables together with stop-based handover and re-boarding mechanisms. Using both simulation-based experiments and a real-world case study, we demonstrate the operational feasibility and cost-effectiveness of the proposed framework under sparse rural transit supply.

To overcome computational complexity, we design a two-stage solution framework—bus-stop clustering followed by an IBKA. IBKA adds opposition-based initialization, random boundary shrinkage, adaptive attack probability, and a differential-evolution hybrid operator, collectively improving search efficiency and convergence. Numerical experiments demonstrate that IBKA surpasses Gurobi, a standard genetic algorithm, and the original BKA in solution quality, stability, and convergence speed, showing strong feasibility on medium- and large-scale instances.

Sensitivity analysis highlights the significant influence of drone range, payload, and bus headway on system performance; high-payload drones prove especially sensitive and thus warrant careful parameter tuning in practice. A real-world case on the Xunyang–Tongqianguan rural line further confirms the model’s practicality: the bus-assisted heterogeneous-drone scheme dramatically reduces total cost compared with conventional truck delivery and outperforms a homogeneous-drone alternative by maintaining feasibility under operating conditions where the homogeneous scheme becomes infeasible, while also achieving a lower total cost in the tested case.

Several avenues remain for future research. First, drone performance and cost parameters in this study are drawn from publicly available specifications and published sources, because comparable drone logistics services are not currently operated in the study area and local operational cost data are unavailable; future work could calibrate these inputs using field measurements or operator-reported data when such services become available. Second, the model assumes perfectly punctual bus departures and does not explicitly represent key operational uncertainties, such as schedule variability, drone failures, and demand fluctuations; incorporating stochastic disruptions and uncertain time windows via simulation-based evaluation, robust optimization, or stochastic programming would improve realism. Third, the current framework relies on two-dimensional Euclidean distances and ignores terrain effects; integrating high-resolution GIS and topographic data could yield more accurate flight paths, travel-time estimates, and executable schedules.

## Supporting information

S1 File Case data and drone parameters. Case dataset and parameter settings used in all experiments.
(XLSX)

S2 File IBKA. Python scripts for the proposed IBKA algorithm.
(TXT)

S3 File BKA. Python scripts for the baseline BKA algorithm.
(TXT)

S4 File GA. Python scripts for the genetic algorithm baseline.
(TXT)

S5 File EGO. Python scripts for the EGO baseline.
(TXT)

S6 File Functions_of_model. Model evaluation functions and auxiliary routines used by all algorithms.
(TXT)

S7 File Run the code all at once. Entry scripts to reproduce the main experiments in one run.
(TXT)
